# Radiation dose comparison of photon-counting breast CT and mammography: A phantom study

**DOI:** 10.1016/j.ejro.2026.100806

**Published:** 2026-08-04

**Authors:** D. Cester, G. Piol, C. Paverd, J. Saenger, D. Bos, S. Shim, A. Boss

**Affiliations:** a Institute for Diagnostic and Interventional Radiology, University Hospital Zurich, University of Zurich, Rämistrasse 100, Zurich 8091, Switzerland; b University of Zurich, Rämistrasse 71, Zurich 8006, Switzerland; c Institute of Diagnostic and Interventional Radiology and Neuroradiology, University Hospital Essen, University Duisburg-Essen, Essen, Germany; d Institute for Radiology and Nuclear Medicine, Regional Hospital Wetzikon (GZO), Switzerland

**Keywords:** Breast cancer, Mammography, Spiral breast CT, Innovative breast imaging, Radiation dose

## Abstract

**Background:**

Spiral breast CT (BCT) systems operate without breast compression while providing more comprehensive three-dimensional information than digital mammography (DM) and digital breast tomosynthesis (DBT). However, before BCT systems can be considered as part of screening programs for breast cancer, concerns about dose exposure in a diagnostic setting (which is generally higher in CT systems) must be addressed.

**Methods:**

This work compares dose measurements between mammography and breast CT with commercial devices and clinical protocols. 3D-printed phantoms of three different sizes were filled with four mixtures simulating different breast densities. Calibrated MOSFET dosimeters measured the entrance surface dose (ESD); in Saddition, a Monte Carlo simulation was performed, matched to the measurements, and used to calculate the average absorbed dose (AAD).

**Results:**

The entrance dose for DM + DBT is always higher than for BCT, up four times for the largest and densier phantom, corresponding to 74% reduction in the ESD for BCT. The results for the AAD vary widely depending on size and density. In the lowest density phantom the dose with BCT is generally higher than with a standard diagnostic mammography protocol (up to +227% increase for the small phantom). However, in case of the highest density, BCT achieved a dose reduction compared to the mammography protocol (-14% for the medium phantom and −15% for the large phantom).

**Conclusions:**

BCT consistently reduced entrance surface dose compared to the combination of one DM and one DBT, while the average absorbed dose was higher for most phantom configurations.

## Highlights

• Breast CT results in higher average absorbed dose than mammography for most clinically relevant phantom configurations.
• Comparable or lower values between breast CT and mammography are observed only at very high glandularity levels.
• Breast CT irradiates all around the breast, resulting in lower surface dose and more homogeneous internal dose distribution.

## 1 Introduction

Breast cancer (BC) remains one of the leading causes of cancer-related mortality among women worldwide [1]. BC screening programs, which can provide BC detection at early stages have been shown to reduce BC-related morbidity and mortality [2], [3]. BC screenings are currently implemented in many countries with different breast imaging modalities [4], including Ultrasound, MRI and X-ray-based imaging. In this work we consider three techniques: two-view digital mammography (DM), digital breast tomosynthesis (DBT) and the recently developed breast CT (BCT).

DM represents the traditional method of breast imaging, a low-dose and well established imaging technique [5]. The main disadvantage of DM is its two-dimensional nature: the superposition of tissues may lead to the hiding of lesions (false negative, loss of sensitivity) or the appearance of non-existing pathologic tissue (false positive, loss of specificity). Moreover, an essential component of DM is breast compression [6], [7]; breast compression optimises image quality and hence visualisation of small lesions by reducing breast thickness, at the price of patient discomfort [8] which contributes to lower re-attendance rates [9]. Another limitation is the reduced sensitivity in dense breasts [10], while the presence of breast implants may further complicate image acquisition with DM [11].

DBT complements DM, it is usually implemented on the same device and it is increasingly being used in diagnostic examinations and screening programs [12], alone or in combination with DM [13]. In DBT, several (from 9 to 25, depending on vendor and model) two-dimensional, low-dose projections are taken at different angles from the longitudinal axis; the image slices are then reconstructed similarly to what happens in CT, resulting in a pseudo 3D imaging technique. This technique has the advantage of reducing the effects due to tissue overlapping and therefore improves sensitivity and specificity [14], [15] however the arc covered by the rotation is only a fraction of the 180° required by full 3D imaging, limiting the z-axis resolution [16]; moreover, the discomfort from compression still represents an issue. Although since a few years devices designed to provide the same exposure between DM and DBT [17], in practice DBT systems in use are on average still associated with higher dose values, as measured in a recent national multicenter, multivendor study [18].

The latest evolution in the field of breast imaging is the introduction of spiral breast CT systems (BCT). BCT systems provide full 3D images with isotropic resolution and completely remove the need for breast compression. In addition to the higher technical complexity of a CT machinery, the additional benefits of tomographic scans are usually balanced by higher levels of patient radiation dose. BCT is not normally included in screening guidelines [4] although the possibility is being explored [19]. While MRI could be considered as a tridimensional alternative to DM and DBT without the use of ionising radiation, BCT excels at detecting ductal carcinoma in situ (DCIS) and microcalcifications in general [20] which is a know limitation of MRI [21]. Recently, the introduction of BCT with photon-counting detectors showed potential to reach low-dose levels similar to DM and DBT [22], [23], [24]; these results are made possible by the reduced pixel size (100µm^2^) and the high absorption efficiency and geometric efficiency, which together result in a higher dose efficiency compared to conventional CT detectors. However the actual level of radiation dose from BCT compared to DM/DBT has not yet been fully investigated with a direct comparison in a clinical setting. A systematic dose assessment for standard-dose diagnostic exams is therefore a necessary step before BCT can be evaluated in the context of BC screening programs.

The objective of this work was to investigate and compare the actual breast dose for a mammography device and a photon-counting BCT in similar conditions, depending on different breast size and density and for two standard diagnostic protocols used in clinical routine.

## 2 Materials and Methods

### 2.1 Devices and protocols

**Fig. 1:**
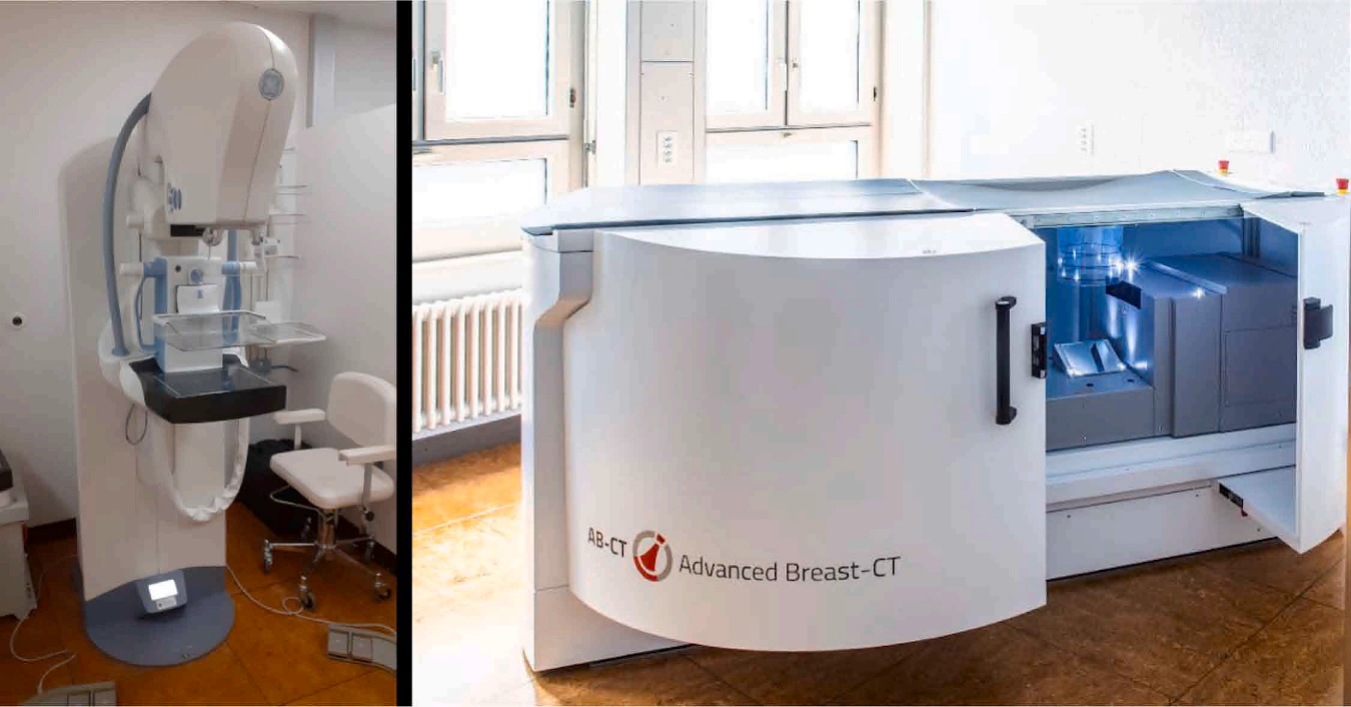
Devices used in this study. Left: conventional mammography (Senographe Essential GE Healthcare). Right: photon-counting spiral BCT (nu:view, Advanced Breast-CT GmbH).

The two compared devices are a conventional mammography unit (Senographe Essential GE Healthcare) and a photon-counting BCT (nu:view, Advanced Breast-CT GmbH), both depicted in Fig. 1. 3D-printed phantoms were repeatedly imaged on both devices for different configurations. In this study the complete diagnostic protocol usedin in our institute (a whole standard diagnostic breast exam) has been compared for both devices.

In general, the most common protocol for mammography exams is composed of two standard views, the craniocaudal (CC) and mediolateral oblique (MLO), both of which can be performed with DM or DBT [25], [26]; in the latter case, a synthetic two-dimensional DM image can be reconstructed from the DBT data with no additional exposure. The standard diagnostic procotol in use in our institute is composed of one DM CC scan followed by a DBT MLO scan (9 low-dose images, equally spaced from −12° to +12°, plus the synthetic two-dimensional reconstruction). In this study we have fully replicated our clinical protocol as a general mammography exam, with one single exception: the phantom design rigidly incorporates the effects of CC compression and therefore our DBT acquisitions have been performed with a CC view instead of the MLO view. Automatic Exposure Control (AEC [27]) was always active and the general scan parameters are reported in Table 1.

**Table 1:** scan parameters for the mammography acquisitions.

Anode / Filter	Rh / Rh
Tube voltage	29 – 31 kVp
Tube current	57 – 62 mA
Exposure time	0.9 – 2.7 s
AEC mode	STD (Standard)
DBT number of projections	9
DBT covered angle	−12° to + 12°
Scan protocol (clinical)	1 DM CC + 1 DBT MLO
Scan protocol (this study)	1 DM CC + 1 DBT CC

For BCT a single spiral scan in High Resolution mode was performed. No system equivalent to AEC is available and the tube current was manually optimized according to our latest recommendations [28] for optimal image quality (Table 2); the tube voltage was fixed at 60 kV.

**Table 2:** *noise-optimized current settings for the breast CT acquisitions. From*[28].

Phantom size	Mixture 1	Mixture 2	Mixture 3	Mixture 4
Large	25 mA	32 mA	32 mA	32 mA
Medium	25 mA	25 mA	25 mA	25 mA
Small	25 mA	25 mA	25 mA	25 mA

### 2.2 Phantoms

We used custom-made 3D-printed phantoms filled with a custom mixture [29] to simulate different patients undergoing breast examinations with both devices. We manufactured a total of six phantoms, with three different volumes (small, medium and large: 247 cm^3^, 640 cm^3^ and 1085 cm^3^, respectively) each in both the compressed and uncompressed version.

**Fig. 2:**
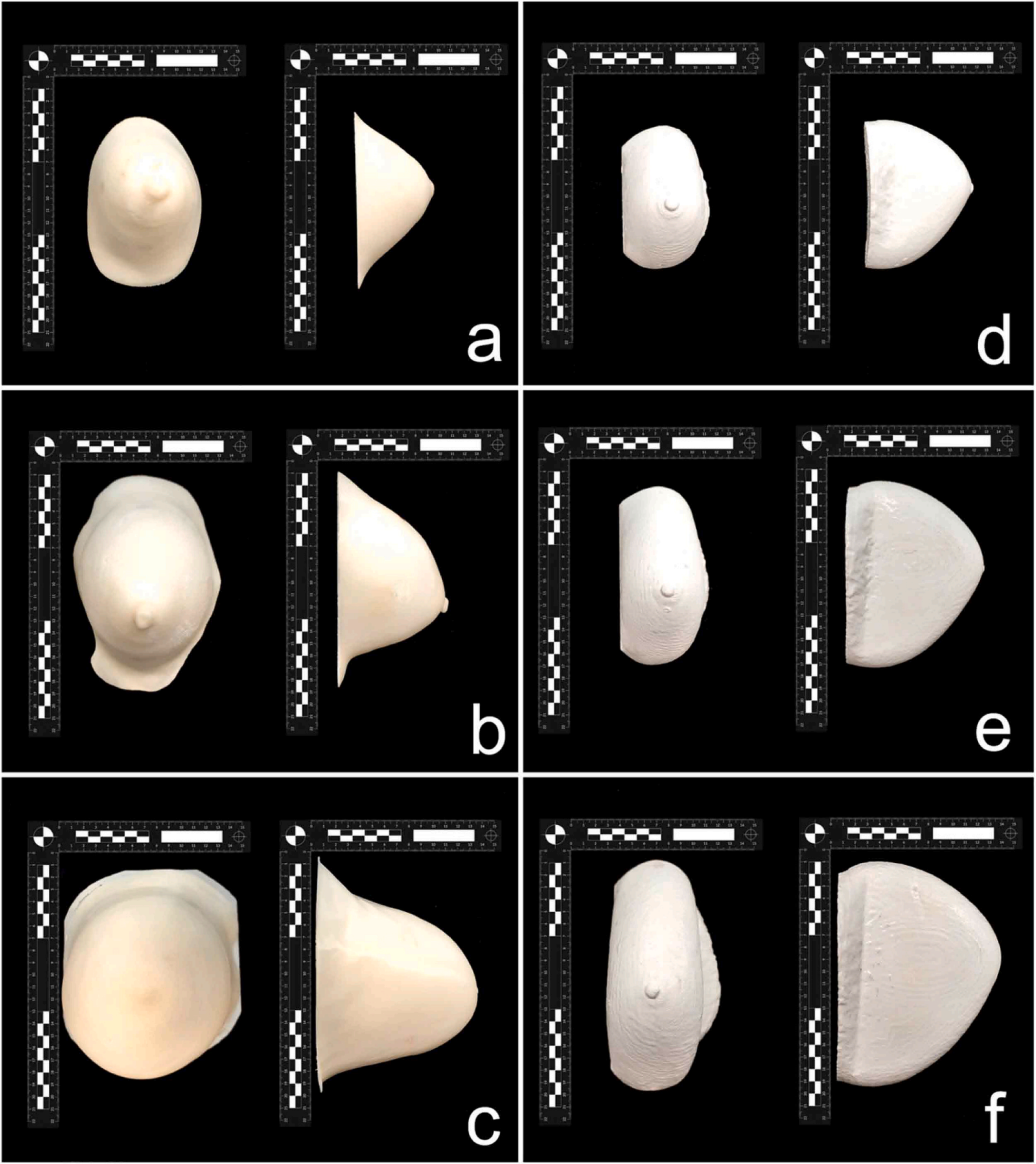
AP and lateral view of the 3D-printed phantoms. a) – c) small, medium and large uncompressed phantoms; d) – f) small, medium and large compressed phantoms.

The breast shapes used to 3D-print the DM/DBT phantoms have been generated with the VICTRE software [30] which included the simulation of the pad compression. The shapes used for the BCT phantoms were extracted from three real datasets having the same volume of the corresponding uncompressed DM/DBT shapes. The phantoms were printed with a Stratasys F370 3D printer (alphacam swiss GmbH) with ABS Ivory; they are shown in Fig. 2.

Four different mixtures of agar solution, rapeseed oil and soy lecythin were prepared in representation of four different categories of breast density [31]. The mixtures have been chosen to be consistent with those used in a previous study [28] and are reported in Table 3; they represent predefined phantom material compositions designed to assess the physical dependence of the investigated dose quantities on glandular fraction, rather than clinically representative volumetric breast-density categories. The fattiest mixture, mainly consisting of pure oil, was not imaged on the breast CT out of concerns for possible spillage; the corresponding measured points are therefore missing, while the simulations based on the virtual shape were unaffected.

**Table 3:** Classification and correspondent composition of the phantom fillings.

Mixture	1	2	3	4
Description	Almost entirely fatty	Scattered tissue	Heterogeneously dense	Extremely dense
Fibrograndular tissue (% vol.)	< 25%	25–50%	50–75%	> 75%
Filling non-fat content • 1.5% Agar solution • Rapeseed oil • Soy lecythin	12.5% 119 ml 831 ml 50 ml	37.5% 356 ml 594 ml 50 ml	62.5% 594 ml 356 ml 50 ml	87.5% 831 ml 119 ml 50 ml
Filling volume (normalized)	1 l	1 l	1 l	1 l
Filling consistency	liquid	gel/liquid	gel	gel

### 2.3 Dose quantities used in the comparison

In the domain of breast imaging three main dose quantities can be used to describe the exposure, all expressed in units of milligray (mGy):

• Entrance Surface Dose (ESD): also called entrance skin dose, this is the dose deposited on the external surface of the patient;
• Average Absorbed Dose (AAD): this is the average dose deposited within the whole irradiated volume;
• Mean Glandular Dose (MGD): this is the average dose deposited within the volume occupied by glandular tissue.

CT devices do not normally report ESD nor MGD; they report the CTDI as average dose, which in contrast to AAD is normalized to a standard reference phantom. A direct comparison of the device-provided values is therefore not possible. In this study we have measured the ESD with the same dosimeters, and also calculated the ESD and the AAD with the same simulation-based method.

The MGD was not included in our comparison. Detailed and validated look-up tables for estimating the MGD from the ESD are available for mammography [32] but the corresponding models are not yet available for BCT; the estimation of MGD via software simulations requires the precise labelling of glandular tissue in the breast model (“segmentation”), however with the VICTRE software it was not possible to control the glandularity of the virtual phantoms with the required precision and therefore only the breast shapes were used.

### 2.4 Measurements with dosimeters

The surface dose measurements have been conducted with real-time MOSFET sensors (BestMedical, TN−1002RD [33]). The MOSFETs were individually calibrated prior to measurement by placing the black surface facing the radiation source, as recommended in the manual. BCT calibrations were performed on a conventional CT scanner using a CT ionization chamber as reference (DCT10, IBA Dosimetry) by exposing them to doses in the range 5 – 40 mGy; mammography calibrations were performed on the same mammography unit using a dedicated mammography dosimeter as reference (Multi Detector XM, IBA Dosimetry) and repeatedly exposing them to doses in the range between 5 and 20 mGy.

The dosimeter readings provided a direct measurement of the ESD for both devices. For the BCT phantoms four sensors were placed circularly around the breast with the same distance to each other (one cranial, caudal, lateral, medial), halfway between the chest-nipple axis. For the mammography phantoms the five dosimeters have been placed along the cranial flat surface of the breast phantoms, also approximately halfway along the chest-nipple axis at a staggered ascending angle.

### 2.5 Monte Carlo simulations

To simulate the irradiation of the breasts, the ImpactMC (AB-CT GmbH) Monte Carlo software was used; this software was developed to reproduce the geometry and physics of the BCT unit and it was adapted to simulate the irradiation from a mammography device. The DICOM data of the actual phantom scans was fed as input for the BCT simulations, while for the mammography simulations the phantom generated with the VICTRE software was used.

As the phantoms were filled with one homogeneous mixture with no internal structure, a single material with the corresponding average density was used in the Monte Carlo simulations.

The Monte Carlo simulations provided a direct estimation of the AAD while the ESD could be obtained by defining ROIs corresponding to the dosimeter placement on the surface of the breast shape. As the tube current in the breast CT is manually set and remains constant during the scan, it is possible to linearly scale the dose values from measurements and simulations. For the validation analysis the breast CT data corresponding to the larger phantom were normalized to the standard current of 25 mA in order to compare our results with the literature reference.

## 3 Results

### 3.1 Measurement validation

To validate the Monte Carlo simulations we compared the measured ESD with the simulated ESD for all corresponding datasets; additionally, we compared the simulated AAD for breast CT and the values extrapolated from a lookup table previously calculated for the same device [28].

**Fig. 3:**
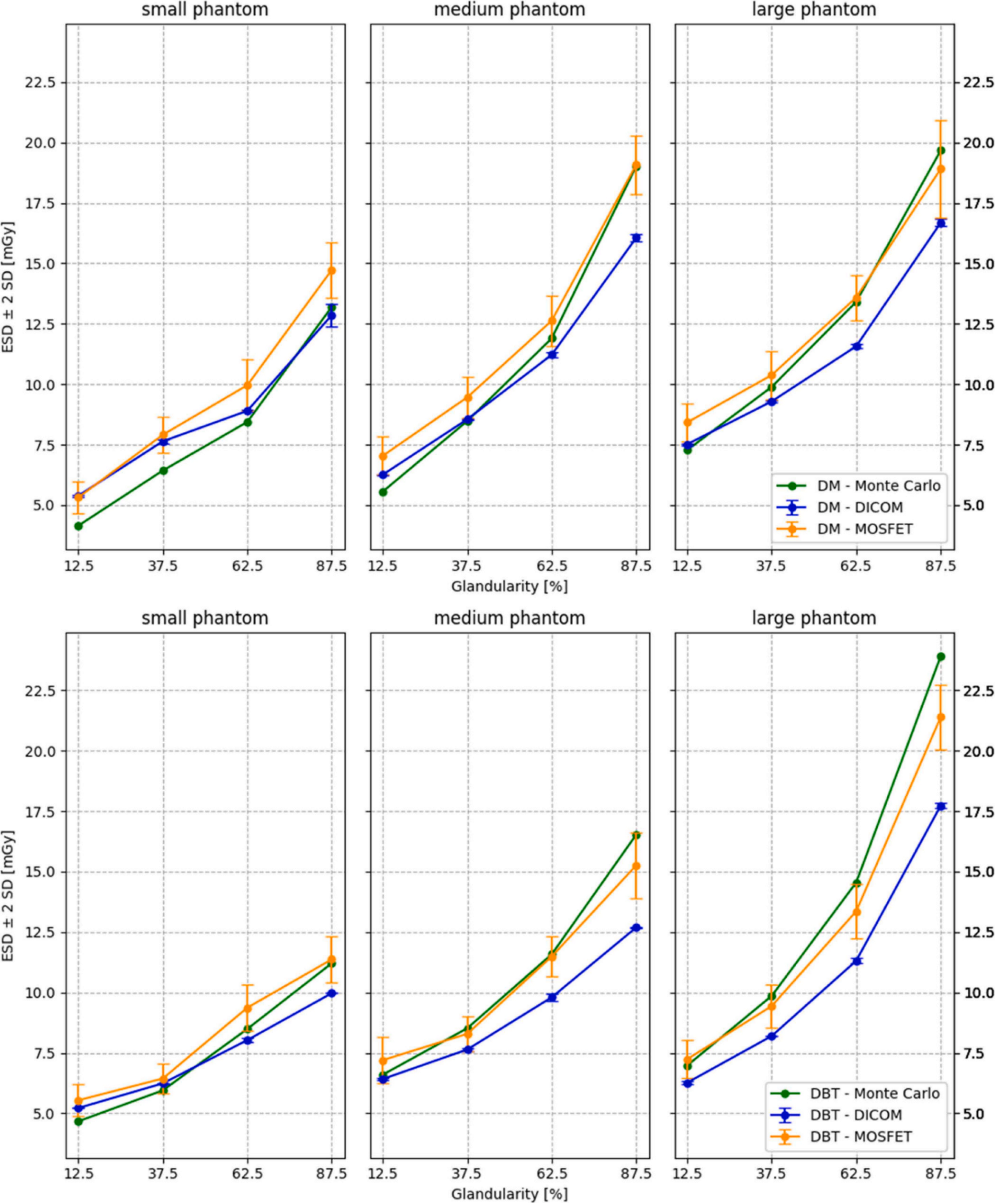
Validation of Monte Carlo Entrance Surface Dose (ESD) values (green line) against the corresponding mammography measurements (orange line), for DM (upper panels) and DBT (lower panels) separately. The values reported by the devices in the DICOM headers are also represented (blue line). The error bars have been set equal to twice the standard deviation.

**Fig. 4:**
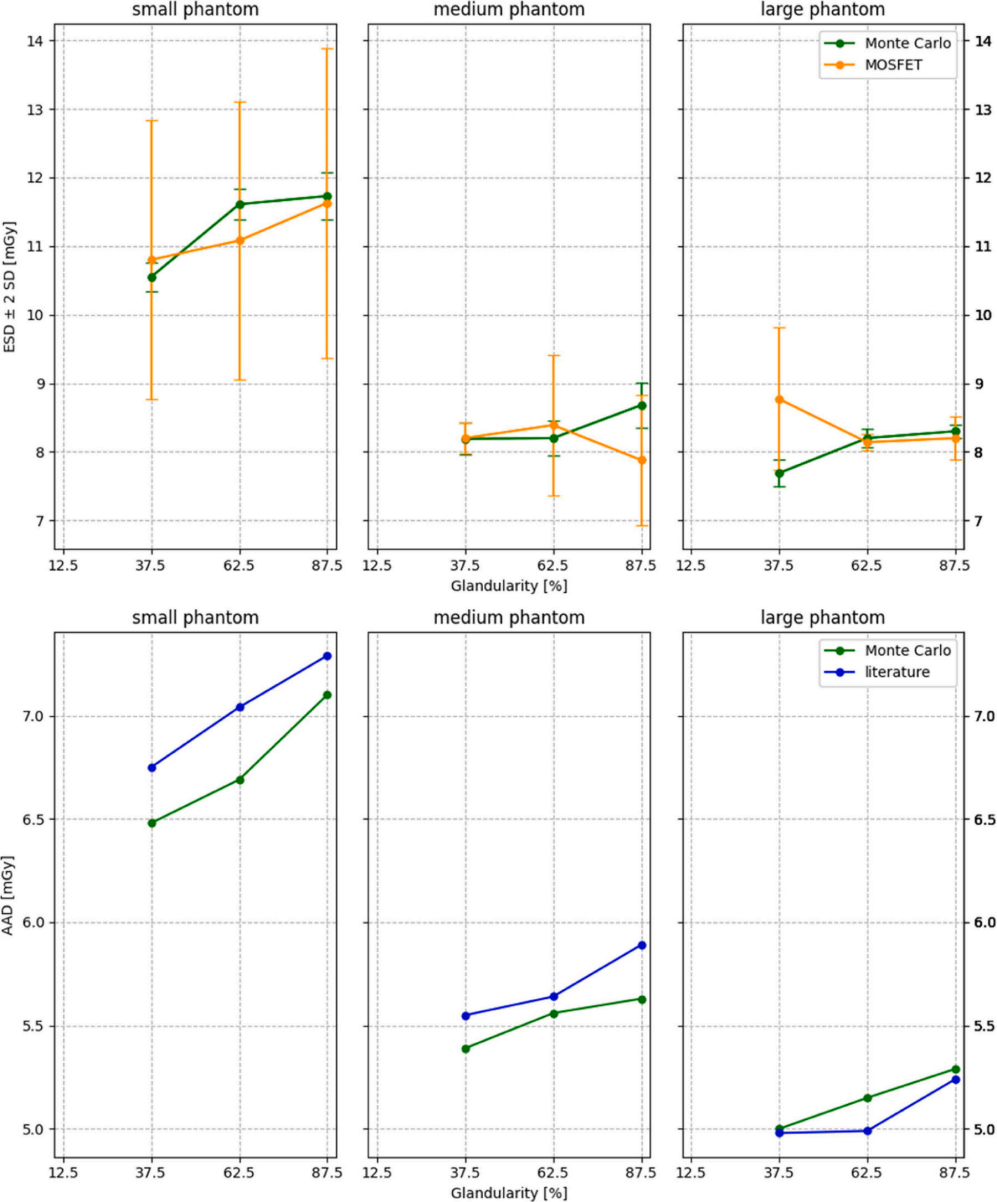
Validation of Monte Carlo Entrance Surface Dose (ESD, upper panels) and Average Absorbed Dose (AAD, lower panels) values against the corresponding reference values for BCT. The Monte Carlo ESD values have been compared with the measured ones, as for DM, with error bars equal to twice the standard deviation. The AAD values have been compared with values from a lookup table (see the text for more details).

The validation data for mammography and for breast CT is reported in Fig. 3 and Fig. 4, respectively. The data shows the expected dependencies of dose on phantom size and density, as well as good agreement in all cases between measurements, Monte Carlo and the previous study. The validation of the Monte Carlo data enabled the use of the simulated AAD values in the subsequent dose comparison.

As an external validation for mammography, the MGD was calculated based on measurement data according to established methods [32] and was found to be compatible with the data collected on several devices [18].

### 3.2 Dose comparison

**Fig. 5:**
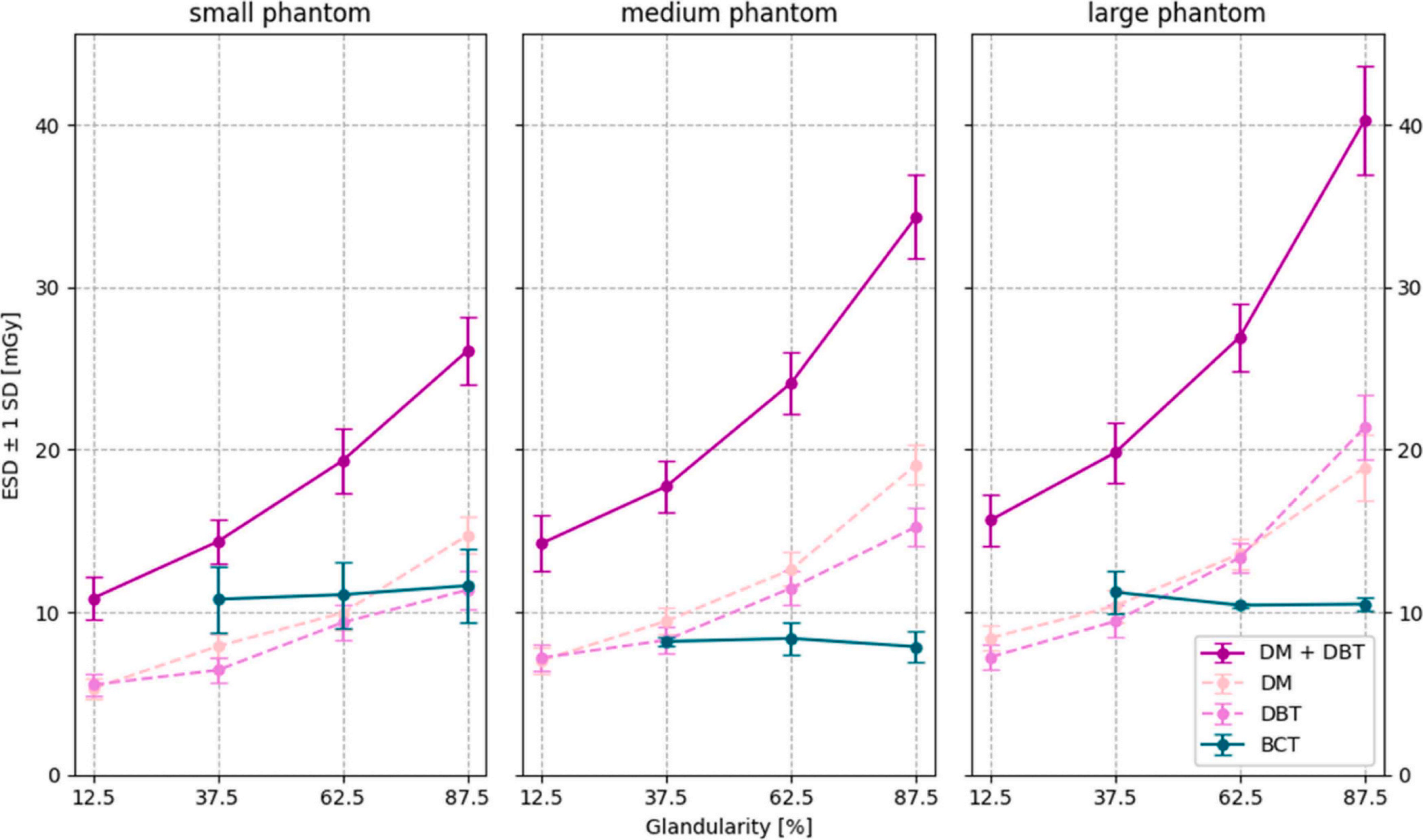
Comparison of measured Entrance Surface Dose (ESD) for a single two-dimensional (DM) or tomosynthesis (DBT) acquisition, a full mammography exam (DM+DBT) and breast CT (BCT), for different phantom size and glandularity. The BCT dose at 12.5% glandularity was not measured for technical reasons (details in the text). The BCT values shown here correspond to the actual tube currents (not normalized to 25 mA). The actual exam dose for mammography corresponds to DM + DBT; the series for the two individual DM and DBT scans have been added for completeness.

The values of the ESD can be directly compared and are shown in Fig. 5, for different phantom sizes and mixture density; the corresponding numerical values are reported in Table 4. The ESD for mammography is always higher than for breast CT, up to a factor of 4 for the largest phantom with the most dense mixture. This corresponds to a 74% reduction in the ESD for the breast CT despite higher current settings (32 instead of 25 mA for the middle and small phantoms).

**Table 4:** Measured ESD for conventional mammography (DM+DBT) and breast CT (BCT). The lowest and highest dose reduction are highlighted in bold.

		**Entrance Surface Dose (ESD)**		
**volume [cm**^**3**^**]**	**glandularity**	**DM + DBT [mGy]**	**BCT [mGy]**	**∆BCT [mGy]**
247	12.5%	10.86 ± 1.31	-	-
247	37.5%	14.35 ± 1.36	10.80 ± 2.03	**−3.55 (−25%)**
247	62.5%	19.33 ± 1.99	11.08 ± 2.03	−8.25 (−43%)
247	87.5%	26.10 ± 2.11	11.63 ± 2.26	−14.47 (−55%)
640	12.5%	14.24 ± 1.74	-	-
640	37.5%	17.75 ± 1.57	8.20 ± 0.23	−9.55 (−54%)
640	62.5%	24.12 ± 1.87	8.39 ± 1.02	−15.73 (−65%)
640	87.5%	34.35 ± 2.57	7.88 ± 0.95	−26.47 (−77%)
1085	12.5%	15.66 ± 1.59	-	**-**
1085	37.5%	19.82 ± 1.88	11.23 ± 1.34	−8.59 (−43%)
1085	62.5%	26.95 ± 2.08	10.43 ± 0.16	−16.52 (−61%)
1085	87.5%	40.30 ± 3.34	10.50 ± 0.40	**−29.80 (−74%)**

The plots and numerical values for the AAD comparison are reported in Fig. 5 and Table 5, respectively. The mammography dose corresponding to a standard protocol lies in the range [1.91, 7.94] mGy while the breast CT dose lies in the range [4.75, 7.10] mGy, depending on the phantom characteristics. For the small phantom the exposure with a breast CT scan is always higher than mammography, from + 51% to + 227%. For the medium and large phantoms the BCT scan also resulted in a higher dose on average, but in case of the highest density phantoms the BCT achieved a dose reduction of −14% and −15% respectively for the medium and large phantoms.

**Table 5:** AAD for mammography Monte Carlo simulations (DM+DBT) and breast CT (BCT) measurements. The highest dose increase and the highest dose reduction are highlighted in bold.

		**Average Absorbed Dose (AAD)**		
**volume [cm**^**3**^**]**	**glandularity**	**DM + DBT [mGy]**	**BCT [mGy]**	**∆BCT [mGy]**
247	12.5%	1.91	6.25	**4.34 (+227%)**
247	37.5%	2.55	6.48	3.93 (+154%)
247	62.5%	3.40	6.69	3.29 (+97%)
247	87.5%	4.70	7.10	2.40 (+51%)
640	12.5%	2.84	5.49	2.65 (+93%)
640	37.5%	3.41	5.39	1.98 (+58%)
640	62.5%	4.81	5.56	0.75 (+16%)
640	87.5%	6.53	5.63	−0.90 (−14%)
1085	12.5%	2.92	4.75	1.83 (+63%)
1085	37.5%	3.89	6.40	2.51 (+65%)
1085	62.5%	5.22	6.59	1.37 (+26%)
1085	87.5%	7.94	6.77	**−1.17 (−15%)**

Fig. 7 shows the internal dose distribution for the two large phantoms with the highest-density mixture, as calculated by the Monte Carlo software. The highest dose deposition is observed in the cranial side of the breast tissue in DM, while the dose distribution in BCT is more uniform and with lower maximum values.

## 4 Discussion

To our knowledge this is the first study directly comparing radiation dose between BCT and DM with DBT using standardized 3D-printed phantoms simulating different breast sizes and densities. All the results refer to the comparison of one diagnostic BCT exam (one 3D scan) with one diagnostic mammography exam (one two-dimensional CC and one MLO tomosynthesis acquisition) as standardized in our institute. We demonstrated that BCT consistently reduced entrance surface dose compared to DM + DBT, and demonstrated an absorbed dose advantage in dense medium-to-large breasts.

From a clinical perspective, the observed dose patterns can be explained by two fundamental technical differences. First, the irradiation geometry differs substantially between modalities. In mammography the irradiation is completely (for DM) or mostly (in case of DBT) coming from the above, while with the spiral BCT the exposure is distributed along the whole surface of the breast; this completely different pattern is clearly visible in Fig. 7. As an immediate consequence of the different distribution of the irradiation around the breast, in the direct comparison of the ESD between mammography and breast CT with our diagnostic protocols, the latter always results in lower values, up to 74% lower for the larger and more dense phantoms. The internal dose distribution is also more uniform for BCT, while the mammography AAD sharply decreases from the upper to the lower side of the breast. Regarding the absolute dose all AAD values for mammography and breast CT exams belong in the single-digit milliGray range. While the AAD is higher with BCT than with DM + DBT for most of the measured sizes and densities, the localized dose in the upper breast region for a single DM CC acquisition always exceeds the highest reported local dose for BCT. This means that the maximum local dose for a standard mammography protocol in our institution, i.e. with both DM and DBT, is higher than with BCT, which is consistent with previous studies [34], [35]. It must be underlined, however, that the lower localized entrance skin dose and the more homogeneous irradiation pattern of BCT do not necessarily imply a reduced stochastic radiation risk, which remains primarily assessed with mean glandular dose or organ-dose estimates. The second technical difference is that mammography systems rely on AEC, which automatically optimizes the tube voltage and current during the scan according to the measured attenuation. Due to the intervention of the AEC, both ESD and AAD exhibit an increase for increasing phantom density and increasing phantom size. AEC systems are also normally implemented in full-body CT systems [36] but are currently not available on the first-generation BCT device used in this study; the tube operates at a fixed voltage and the current is manually selected by the device operator and kept constant during the scan. The local recommendations for current selection are based on a previous optimization study [28] however only a subset of values is selectable from the interface and no modulation during the scan is possible, forcing the recommendations to represent a conservative choice based on the thickest section of the breast. As a consequence, the ESD and AAD values for BCT are confined to a narrower range, with less pronounced dependence on the phantom size and density than DM/DBT, as visible in Table 4 and Table 5. The implementation of AEC on different devices resulted in substantial variations in radiation dose optimization [37], [27]; analogously, the full implementation of an AEC system for the breast CT device carries the potential for a significant reduction of the administered dose. Even without the benefits of a fully automated AEC (which in all cases should reduce the dose compared to the conservative, manually selected values used in the system at present) breast CT simulations resulted in lower exposure for medium and large phantoms with high density.

Given that the most difficult population subset for mammography breast screening is the dense breast (high-dose) category [38], the potential for dose reduction in this category is of particular relevance. Several studies have demonstrated that women with dense breast tissue receive higher mean glandular doses during mammography. In the recent review by Moda et al. increased mammographic breast density was consistently identified as a significant determinant of mean glandular doses, largely mediated by greater compressed breast thickness and higher exposure parameters required to adequately penetrate fibroglandular tissue [39]. In a multivariate analysis higher mean glandular doses was associated with a 1.4-fold increase in cancer risk, reflecting its contribution to increased radiation dose [39]. The potential for dose reduction associated with BCT was initially described while the technology was being proposed [34]; further studies performed on research devices substantiated the possibility to match [35] or even reduce [23] the patient dose compared to mammography. By means of a direct comparison between commercial devices with standard protocols, our study confirms the possibility of achieving with BCT dose values of the same order of magnitude (mGy) as mammography in a clinical setting. Similar or even lower dose levels were observed only for extremely dense phantom; these high-glandularity configurations should be interpreted cautiously as they exceed the density normally found in a clinical setting. Given the ongoing public concern regarding radiation levels in mammographic imaging, it is essential to provide clear and transparent information about radiation exposure to patients and medical personal, including the comparative dose for each examination [39].

Besides the increased risks due to radiation compared to the general population and the high risk of masking breast cancer [40], women with extremely dense breasts in the screening age range have also an increased risk of developing breast cancer, which is approximately twice as high as for the ‘average’ woman, and almost 4–6 times as high as in women with almost entirely fatty breasts [41]. Furthermore, dense breast tissue has also been associated with increased discomfort and pain during mammographic compression, which may negatively affect patient acceptance [39]. In this context, and particularly for women with dense breasts, the combination of tridimensional tomographic imaging without tissue overlap and the absence of breast compression with BCT may represent an additional, patient-centered advantage which should be weighted against the risk associated with the corresponding dose values.

### 4.1 Limitations

This study has multiple limitations. First, the phantom size and mixture compositions were chosen to allow the investigation of the broad physical dependence of the investigated dose quantities on volumetric glandular fraction, as well as the correspondence with previous works; subsequent studies should focus on the actual sizes and densities that are mostly representative of the actual patient population. Second, the most relevant parameter associated with risk of breast cancer is the MGD, which calculation was not easy to incorporate in the study, limiting our results to a direct comparison of ESD and AAD; conclusions regarding clinical radiation risk should be interpreted with caution in the absence of precise MGD estimates.

**Fig. 6:**
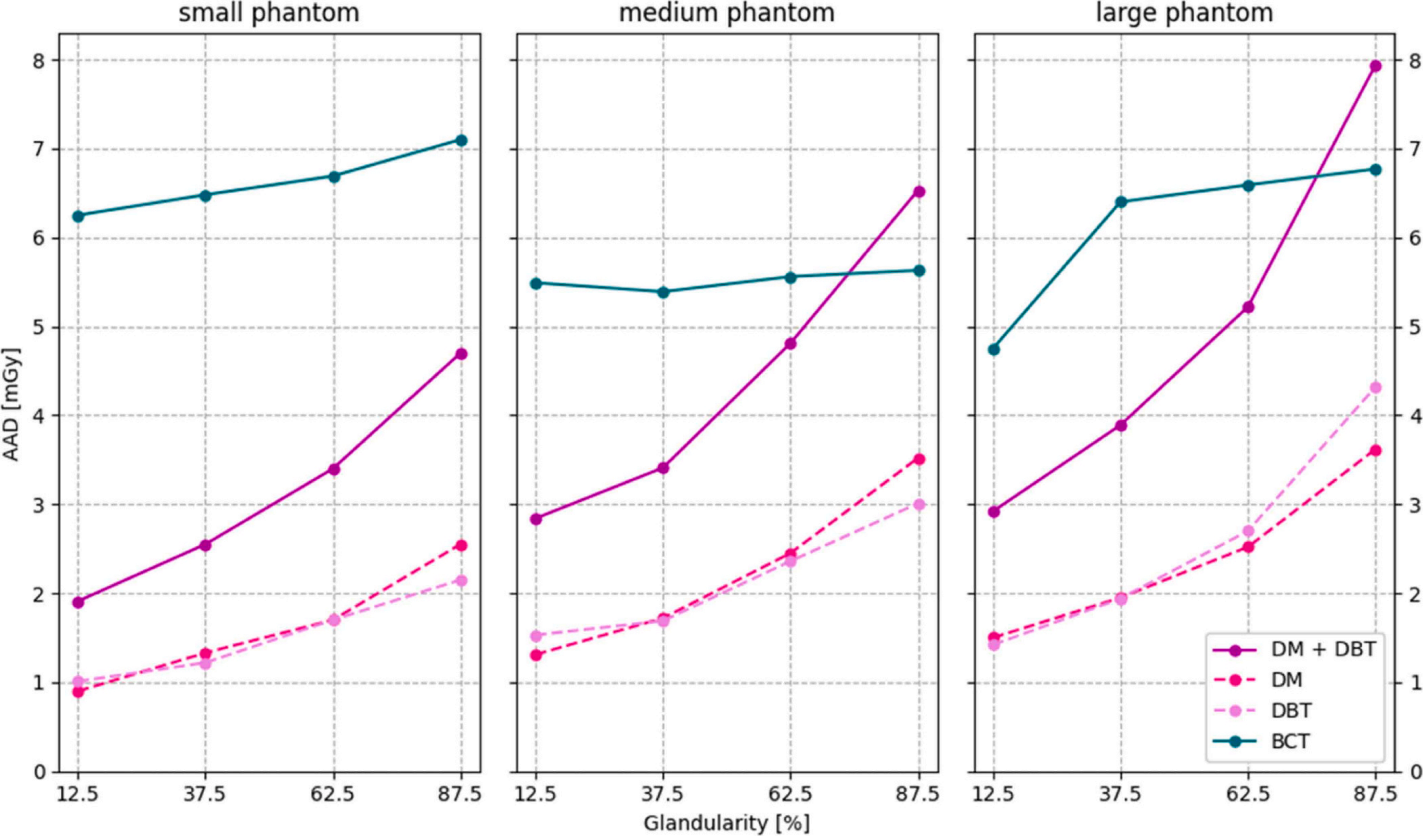
Comparison of simulated Average Absorbed Dose (AAD) for a single two-dimensional (DM) or tomosynthesis (DBT) acquisition, a full mammography exam (DM+DBT) and breast CT (BCT), for different phantom size and glandularity. Error bars are not present since the reported uncertainty of the Monte Carlo simulations is negligible. The BCT values shown here correspond to the actual tube currents (not normalized to 25 mA). The actual exam dose for mammography corresponds to DM + DBT; the series for the two individual DM and DBT scans have been added for completeness.

Moreover, this study was limited to two devices available at our institute, and the comparison was based exclusively on the two clinical protocols currently in use; the results constitute a baseline for dose comparison but the dose optimization potential for different devices, clinical protocols and algorithms should be further investigated, as the actual choice of projections and their respective dose could differ depending on country, institute and device model [18]. Due to technical constraints we replaced the DBT-MLO scan with a DBT-CC. It must be noted that DBT-MLO scans result on average in a slightly higher exposure than DBT-CC [17], [18], [42] and therefore our results for mammography underestimate the exposure from the clinical protocol; however this difference can be considered small compared to inter-patient and -device variability [18] and therefore a detailed correction was not incorporated in the numerical analysis. Relatively small differences were also observed between DM CC and DBT CC (Fig. 5 and Fig. 6), in agreement with the vendor [17], and therefore we have not explicitly analyzed DM + DM or DBT + DBT protocols; the applicability of the results obtained with our DM + DBT protocol to other institutional settings, while generally possible, may depend on the characteristics of the devices being considered. In particular, workflows with only DBT images complemented by synthetic 2D images (DBT+s2D) are increasingly being performed; these workflows are not equivalent to the investigated DM + DBT and given the variability of DBT parameters (number of projections, angle, dose per projection), the translation of our findings should be appropriately analyzed in the context of the specific protocols and devices actually in use.

**Fig. 7:**
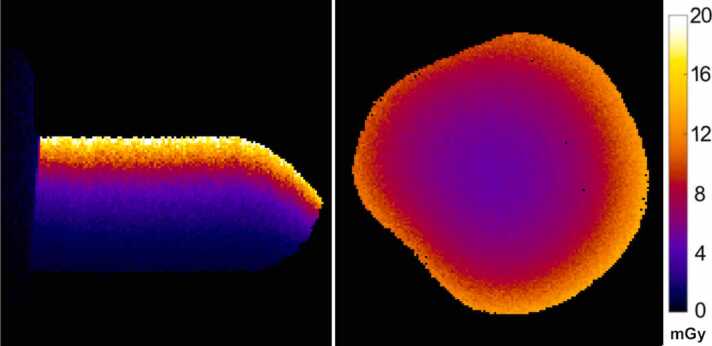
Monte Carlo comparison of the internal dose distribution between DM (left panel, trasversal view, CC acquisition irradiated from the top, thickness 6.1 cm) and breast CT (right panel, axial view, irradiated from all sides, average diameter approx. 11.5 cm). Both distributions correspond to the large phantom size (1085 cm^3^) and the mixture representing 62.5% glandularity.

Finally, due to technical limitations at this stage of the phantom development it was not possible to place the dosimeters within the filling and therefore the direct measurements were limited to the surface dose.

## 5 Conclusion

Our findings show the respective AAD dose values for matched phantom measurements between breast CT and mammography with clinical diagnostic protocols, with breast CT resulting in higher AAD values than mammography for most clinically relevant phantom configurations, while lower or comparable values are observed only at very high glandularity levels. Additionally, we describe the lower localized ESD and the more homogeneous irradiation pattern observed with BCT. Our data can be used in the evaluation of BCT as an imaging option for diagnostic applications and serve as reference for further evaluation in patient-based studies.

## Ethics in Publishing Statement

All authors agree that:

This research presents an accurate account of the work performed, all data presented are accurate and methodologies detailed enough to permit others to replicate the work.

This manuscript represents entirely original works and or if work and/or words of others have been used, that this has been appropriately cited or quoted and permission has been obtained where necessary.

This material has not been published in whole or in part elsewhere.

The manuscript is not currently being considered for publication in another journal.

That generative AI and AI-assisted technologies have not been utilized in the writing process or if used, disclosed in the manuscript the use of AI and AI-assisted technologies and a statement will appear in the published work.

That generative AI and AI-assisted technologies have not been used to create or alter images unless specifically used as part of the research design where such use must be described in a reproducible manner in the methods section.

All authors have been personally and actively involved in substantive work leading to the manuscript and will hold themselves jointly and individually responsible for its content.

## Funding statement

This project has been supported by Swiss Cancer Research Foundation grant KFS−5524–02–2022.

## Declaration of Competing Interest

The authors declare the following financial interests/personal relationships which may be considered as potential competing interests: Andreas Boss reports financial support was provided by Cancer Research Foundation Switzerland. If there are other authors, they declare that they have no known competing financial interests or personal relationships that could have appeared to influence the work reported in this paper.
